# Epidemiological Investigation of Feline Upper Respiratory Tract Infection Encourages a Geographically Specific FCV Vaccine

**DOI:** 10.3390/vetsci10010046

**Published:** 2023-01-09

**Authors:** Jindong Gao, Yan Li, Qiyun Xie, Mayasar I. Al-zaban, Fatimah A. Al-Saeed, Ali A. Shati, Amin A. Al-Doaiss, Ahmed Ezzat Ahmed, Shah Nawaz, Hala Ebrahem, Irfan Irshad, Muhammad Fakhar-e-Alam Kulyar, Jiakui Li

**Affiliations:** 1 College of Veterinary Medicine, Huazhong Agricultural University, Wuhan 430070, China; 2 Department of Biology, College of Science, Princess Nourah bint Abdulrahman University, P.O. Box 84428, Riyadh 11671, Saudi Arabia; 3 Department of Biology, College of Science, King Khalid University, Abha 61413, Saudi Arabia; 4 Department of Theriogenology, Faculty of Veterinary Medicine, South Valley University, Qena 83523, Egypt; 5 Pathobiology Section, Institute of Continuing Education & Extension, University of Veterinary and Animal Sciences, Lahore 54000, Pakistan; 6 Department of Animal Nutrition and Feed Science, College of Animal Science and Technology, Huazhong Agricultural University, Wuhan 430070, China; 7 College of Animals Husbandry and Veterinary Medicine, Tibet Agricultural and Animal Husbandry University, Linzhi 860000, China

**Keywords:** feline upper respiratory tract, feline calicivirus, feline herpesvirus-1 type, Mycoplasma felis, Chlamydia felis, PCR, epidemiological investigation

## Abstract

**Simple Summary:**

Feline upper respiratory infection (FURI) is a frequent ailment in felines. It feels like a cold, but it has the potential to be far worse. Such infections in cats are most often caused by viruses, perhaps between 80% and 90%, while bacteria are responsible for the remaining 10% or so. Clinical sign analysis revealed that *feline calicivirus* (FCV) infections were most often linked with oral symptoms, whereas *feline herpesvirus* (FHV) infections were most often consorted with sneezing. It is the first research to the authors’ knowledge that provides epidemiological results of FURI in cats in Wuhan region of China. Every veterinarian may benefit from the outcomes described, since doing so will refresh their understanding of FURI. Anticipating the development of a more phylogenetically similar FCV vaccine, we discovered that the strains connected with the F9 and 255 vaccines were distant, which may lead to vaccination failure. Hence, it is necessary to encourages geographically specific FCV vaccine.

**Abstract:**

A total of 1158 cats with feline upper respiratory tract infection were incorporated from twenty animal hospitals in Wuhan, China, from April 2019 to April 2022 to investigate the epidemiology of *feline calicivirus* (FCV), *herpesvirus-1* (FHV-1), *Mycoplasma felis* (*M. felis*) and *Chlamydia felis* (*C. felis*) for the development of a geographically-specific FCV vaccine with reference to prevalence and risk factors for infection. The 871 samples (75.2%) of kittens were younger than 12 months, of which 693 were males, and 456 were females. Among the samples, 443 were British shorthair cats, accounting for 38.3%, and 252 were Chinese rural cats, accounting for 21.8%. PCR/RT-PCR detection of the above four viruses (FCV, FHV-1, *M. felis*, and *C. felis*) in the upper respiratory tract of cats showed that the total positive samples were 744 (64.3%), including 465 positive samples of *feline calicivirus*, accounting for 40.2% of the total 1158 samples. There were 311 positive samples of *M. felis*, accounting for 26.9% of the total samples, ranked second in clinical practice. The 180 positive samples of *feline herpesvirus* accounted for 15.5%, and 85 positive samples of *Chlamydia felis* accounted for 7.3%. Among them, the number of positive samples of single pathogenic infections was 493, accounting for 66.3% of the total 744 positive samples. Double, triple, and quadruple infections accounted for 28.2%, 5.0%, and 0.5%, respectively, with the highest proportion of single infections. The molecular biological characteristics of the 17 isolated FCVd strains in Wuhan were further analyzed. It was found that the F9 vaccine strain and the antigenic epitopes in the 5’HVR of the E region were collated with the F9 vaccine strain. Moreover, phylogenetic tree analysis showed that the strains related to the F9 and 255 vaccines were distantly related, leading to the failure of the vaccine. In addition, the strains associated with the F9 and 255 vaccines were distant, which might lead to vaccine failure in anticipation of the development of a more phylogenetically close FCV vaccine in China and may require the development of a vaccine for a locally related FCV strain.

## 1. Introduction

Several viral and bacterial pathogens, including feline *herpesvirus-1* (FHV-1), *Mycoplasma felis* (*M. felis*), *Chlamydia felis* (*C. felis*) and *bordetella bronchiseptica* (*b. bronchiseptica*), enter the cat’s respiratory system and cause upper respiratory tract infections [1]. The FHV-1 is a double standard DNA virus, present globally with no zoonotic importance, unlike other herpes viruses, e.g., *Cercopithecine herpesvirus-1* [2]. FCV is a single standard RNA non-enveloped virus with vast antigenic variation in a single serotype. Infected cats with FCV may even shed the virus for life [3,4]. *C. felis* is an obligate intracellular bacterium that can only survive within the host. Initially, it causes unilateral signs of disease in infected cats but may result in bilateral ocular infection [3,5]. *M. felis* is a pleomorphic gram-negative bacterium that causes feline upper respiratory tract infections. According to some studies, mycoplasma is a normal resident of the upper respiratory tract [6,7,8], playing a direct or indirect role in conjunctivitis and upper respiratory tract infections [9].

Upper respiratory tract conditions, which include conjunctivitis, are a frequent complication in cats, including those found in breeding catteries, shelters, and household cats [10,11]. Major causes of URTD in cats include *feline calicivirus* (FCV) and *feline herpesvirus-1* (FHV). The World Small Animal Veterinary Association classifies immunizations against FHV and FCV as core vaccines, and many cats receive their shots regularly. These vaccines reduce clinical symptoms, but do not prevent clinical symptoms [12]. *Feline herpesvirus* and FCV have been considered equally relevant in feline respiratory diseases, but new research reveals that FCV may be more frequent. Clinically recovering cats may become carriers for both viruses. FCV carriers shed continually, but FHV carriers occasionally shed, with the virus remaining latent most of the time. Vaccines are available that provide reasonable disease protection but do not prevent infection or the emergence of the transmission cycle [13]. Hence, upper respiratory tract infections in cats are very common in animal hospitals and boarding facilities, especially in young cats. They cause sneezing, runny nose, conjunctivitis, eye secretions, oral gums, tongue ulcers, and/or coughing [14,15]. *Chlamydia psittaci* was the first identified respiratory pathogen in cats and is thought to be responsible for most cases of respiratory disease along with *feline herpesvirus-1* (FHV-1) and *feline calicivirus* (FCV). Feline upper respiratory infections are more common in outdoor and household cats or cats with a travel history [16]. These infections lead to respiratory disease and are a major concern for veterinarians, cat owners, and shelter operators. Moreover, morbidity far exceeds mortality, but in young kittens, mortality may also increase. Such exposure to disease at shelters can discourage stray cat adoption. Moreover, the costs associated with treatment and prevention are important for a shelter’s functionality[17].

Nowadays, only one clinically licensed vaccine for immunization against feline panleukocyte syndrome, *feline calicivirus*, and herpes virus is used in China. Typically, the positive FCV in fully vaccinated cats (Rhinotracheitis–Calici–Panleukopenia killed vaccine by Zoetis, USA) with upper respiratory tract infections is ranged from 23.46% to 87.5%. Some previous research noticed the same range in Beijing, Hangzhou, Nanning, Shanghai, and Southwest China [11,12]. While in a city such as Wuhan, China, where most of the population love to have cats as pets, there is no such record. So, this urged us in the following study to find a geographically-specific FCV vaccine concerning prevalence and risk factors for infection. Such data would provide a basis and theoretical reference for developing a specific-strain base vaccination to prevent and treat upper respiratory tract infections in cats all over the country.

## 2. Materials and Methods

### 2.1. Source of Samples and Collection Area

In this study, 1158 cats’ ocular, nasal and oropharyngeal samples with clinical signs of feline upper respiratory tract infection were collected with the help of cotton swabs from twenty animal hospitals in Wuhan, China, during the period from April 2019 to April 2022. Different centers were selected to collect samples from all the hospitals within a 1 kilometer radius of each point of the study region. The cats mainly suffered from upper respiratory tract infections and had clinical symptoms, e.g., conjunctivitis, sneezing, coughing, oral ulcers, and increased nasal and ocular secretions.

### 2.2. Ethical Approval and Owner Consent

The ethical committee of Huazhong Agricultural University approved the study. Moreover, the owners of the cats were aware of the sampling before collection.

### 2.3. Viral DNA Extraction

All collected samples were dissolved using sterilized phosphate buffered saline (PBS). DNA was extracted from samples using a Simply PVirus DNA Extracting Kit (BSC67; Bioer, Hangzhou, China). The kit used a unique lysis buffer of Bioer and special polymer membrane material. According to the manufacturer’s instructions, viral RNA was extracted using Total RNA Extraction Reagent (Vazyme, Nanjing, China). The purity and concentration of the RNA were determined using gel electrophoresis and the Thermo Fisher Scientific, China, Nanodrop 2000 analyzer at 260 and 280 nm by following the Kulyar et al. [18]. The absorption ratios (260/280 nm) of all tested samples ranged between 1.8 and 2.3. HiScript II 1st Strand cDNA Synthesis Kit (Vazyme, Nanjing, China) was used for 1 μg of total RNA to reverse transcription, following polymerase chain reaction with 20 μL of RT reaction products after incubation (42 °C, 2 min; 37 °C, 15 min; 85 °C, 5 s).

### 2.4. PCR/RT-PCR Detection

FHV-1 (TK gene), M. felis (28S gene), C. felis (OMP2gene) primers were synthesized by referring to previous studies (1–3) (Table 1). VP2 gene of FCV primers was designed according to Accession No. MW658467. Primer sequences are shown in Table 1. According to the manufacturer’s instructions, 2 × Phanta Max Master Mix and HiScript II One Step RT-PCR Kit (Vazyme, China) were used to amplify DNA or RNA of the samples. The reaction conditions were as follows: reverse the RNA to cDNA at 50 °C for 30 min, pre-denaturation at 95°C for 5 min, amplification phase at 95 °C for 30 s, 55 °C for 30 s, 72°C for 45 s, extension for 35 cycles, and final extension at 72 °C for 5 min. Then we performed sequencing of positive samples. All details concerning the FCV isolates sequencing were submitted to the GenBank nucleotide database (GeneBank No. OP904199- OP904215)

### 2.5. Histopathological Analysis

Histopathological analysis was performed to check the stomatitis level, as it is important to study the mechanism caused by FCV in cats. For this purpose, the necrotic oral tissue from confirmed positive cases was fixed in formalin and embedded in 10% neutral paraffin blocks. Histopathological analyses were performed to compare the feline upper respiratory tract infection changes. For this purpose, the sections with a thickness of 4–5 micrometers were manually sectioned using a microtome after being fixed. Dewaxed sections were stained with hematoxylin and eosin (H&E). Many stages and procedures were required to provide standard and interpretable sections by adopting the protocol by Slaoui and Fiette [23]. Finally, the histopathological slides were dehydrated and sealed for microscopic examination.

### 2.6. Nucleotide (nt) and Amino Acids (AA) Homology Comparison Analysis

The 17 FCV VP1 genes and 34 reference strains isolated in this study were analyzed using the Lasergene v7.1 (https://www.dnastar.com/software/lasergene/) (accessed on 13 September 2022)software analysis package. By comparing the Clustal W method in MegaAlign, the sequence distance was obtained for further calculation of the homology range.

### 2.7. Amino Acid Site Mutation Analysis

Following the previous methodology, the MegaAlign for Clustal W method comparison and Alignment decoration in Alignment Report was used to analyze the amino acid site mutation.

### 2.8. Phylogenetic Tree Analysis

Using Mega v11.0.10 (https://megasoftware.net/) (accessed on 24 September 2022) an optimal nucleotide substitution model was selected. The maximum likelihood phylogenetic tree was established, and visual output analysis was performed.

### 2.9. Statistical Analysis

IBM SPSS Statistics (20.00) was used to conduct the statistical analysis. Chi-square test and univariate ordinary logistic regression analysis were used to evaluate the univariate correlation. The significance level was considered at the probability value of <0.05.

## 3. Results

### 3.1. Epidemiological Investigation

**Figure 1:**
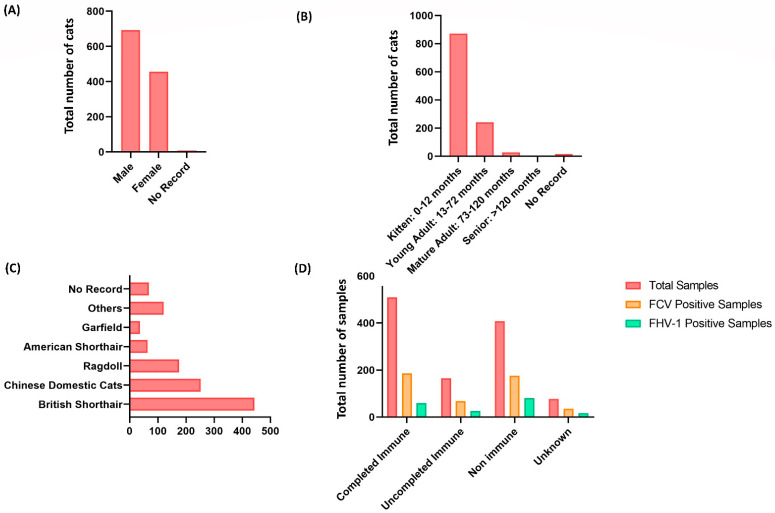
Distribution of upper respiratory tract infection in 1158 collected samples of cats living in Wuhan, China. (**A**) The gender distribution of cats with upper respiratory tract infection; male cats account for a higher proportion than female cats. (**B**) The age range of cats suffering from upper respiratory tract infections. The younger cats suffered the most, notably those aged 0 to 12 months (871 out of 1158). (**C**) The distribution of breeds of cats with upper respiratory tract infections. British shorthair cats account for the highest number of cases (443 out of 1158). (**D**) Immunization status of cats with upper respiratory tract infection, revealing a more significant frequency of infected cats with complete viral immunity.

Statistics of different genders, ages, breeds, living environments, immune status, and onset symptoms of the 1158 collected samples were evaluated. According to the distribution of different genders, male cats accounted for a larger proportion of upper respiratory tract infections (Figure 1A). The age distribution was noticed to be more prevalent in younger cats, especially 0–12 months old cats, accounting for the highest proportion, reaching 871 cases (Figure 1B). According to the statistics on breeds, the British shorthair cats accounted for the most significant proportion out of the breeds, with 443 cases (Figure 1C). Even though a greater number of these infected cats demonstrated complete immunity to the virus (Figure 1D). However, it is essential to note that the absence of any detectable harmful microbe infection does not imply full immunity [24].

**Figure 2:**
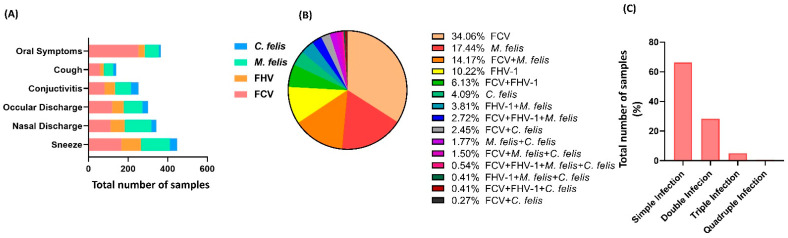
Different characteristics of upper respiratory tract infection in cats living in Wuhan, China. (**A**) Clinical symptoms of cats with upper respiratory tract infection. *Mycoplasma*, *feline herpesvirus*, and *feline calicivirus* mainly cause sneezing. (**B**) Distribution of pathogenic microorganisms of cats with upper respiratory tract infection. The higher rate of microorganisms was reported as 34.06% for FCV infection and 65.94% overall (*p* < 0.05). (**C**) Percentile distribution of cats’ samples based on infection intensity. Simple infection accounted for more than double, triple, and quadruple infections (*p* < 0.05).

Based on clinical symptoms, feline calicivirus infection mainly had oral symptoms, feline herpesvirus and mycoplasma had mainly sneezing, and feline chlamydia had conjunctivitis (Figure 2A). The incidence of FCV, FHV-1, M. felis, and C. felis infection in different seasons was counted (Table 2). The overall positive detection rate of infection was 64.3%. The infection rate of FCV was the highest (40.2%), while the detection rate of infection in autumn was the highest, accounted 66.9%. However, there was no statistical significance compared to other seasons (*p* > 0.05). Statistics on the infection of various microorganisms were carried out separately (Figure 2B). The infection rate of FCV was the highest, reaching 260 cases (128 cases of M. felis and 104 cases of mixed infection) of FCV and M. felis. The infection rate of a single microorganism was 65.6%, and the mixed infection rate was 0.4% from the perspective of microbial infection (Figure 2C).

### 3.2. Histopathological Analysis

**Figure 3:**
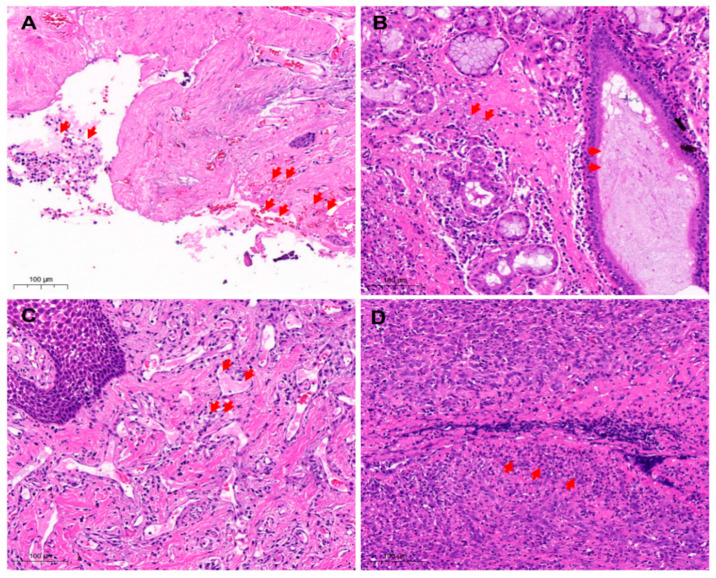
Histopathological analysis of oropharyngeal samples. (**A**) Loss of oral mucosa (H&E 20×). The gingival mucosa epithelial cells fell off, and the lamina propria was congested and hemorrhaged with many inflammatory cells that targeted endothelial cells (arrows) and contributed to the developmental abnormalities of structure and function. (**B**) The mucosal epithelial cells were necrotic and exfoliated (H&E 20×). The inflammatory cells were extensively infiltrated and necrotic. The acinar epithelial cells degenerated, filling the acinar cavity with mucus (arrows). (**C**) Necrosis of cells in the lamina propria of gums (H&E 20×). The lamina propria was hyperemic, thrombus appeared in the blood vessels with the extensive infiltration of inflammatory cells (arrows). In addition, nuclear condensation, nuclear fragmentation, and nuclear lysis were noticed. (**D**) Oral tissue cell necrosis (H&E 20×). Bleeding, congestion, infiltration of inflammatory cells (arrows), and necrosis of the inherent layer of the gums. Red arrows mark hemorrhage, shedding of inflammatory cells, thrombosis, and necrosis of inflammatory cells.

The histopathological observation showed degeneration, necrosis, and shedding of gingival mucosa epithelial cells with lamina propria hemorrhages (Figure 3A). The blood vessels became engorged with blood clots (Figure 3B). Moreover, a large number of inflammatory cells were infiltrated and necrotic. The acinar epithelial cells were swollen and necrotic. Additionally, the acinar cavity was filled with mucus (Figure 3C,D).

### 3.3. Comparison of Nucleotide and Amino-Acid Homology between FCV Strains

The comparison of nucleotide (nt) and AA homology between 17 *FCV* isolates in this study and 34 reference strains showed that the nt and AA homology between the isolates and the reference strains were 67.3–84.4% and 90–96.7%, nt and AA homology between isolates were 68.3–98.4% and 90.9–99.2%, respectively (Table 3). Moreover, the similarity between F9 and 255 strains were AA: 93% and nt: 73.8%, respectively.

### 3.4. The Amino Acid Site Mutation Analysis

**Figure 4:**
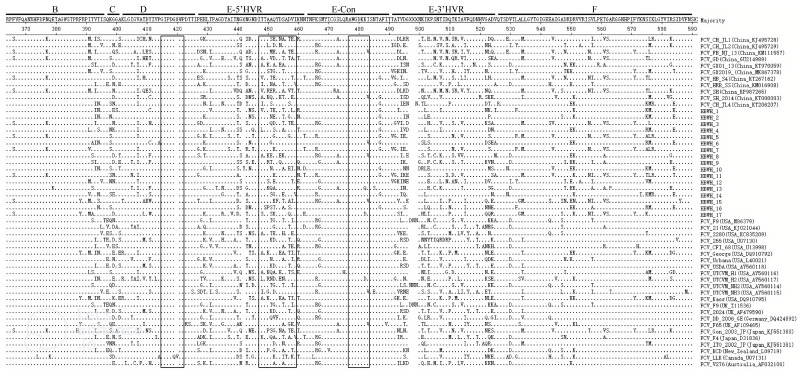
Amino acid site mutation analysis of isolates and reference strains performed in the ORF2 region (B–F) of the capsid protein gene proposed by Seal [25]. (Inside the box is the epitope region of the VP1 gene).

The amino acid site mutation analysis of the 17 isolates is shown in Figure 4, including B (364–396 AA), C (397–401 AA), D (402–426 AA), E (427–525 AA), and F (526–590 AA) of the VP1 region of ORF2 (526–590 AA). Important immune-related targets of FCV are located in the VP1 region of ORF2, which are located in the D, E-5’HVR, and E-Con regions (boxed) [22]. The results show that the FCV isolates in this study have relatively conservative antigens in the D and E-Con regions. Only the I483V mutation appears in the E-Con region. It is worth noting that in the antigen in the E-5’HVR region, there were more complex changes in epitopes (447–459), which were different from F9-vaccine-associated strains (GenBank No. M86379). These may lead to changes in antigenic epitopes, resulting in immune failure (Figure 4).

### 3.5. Phylogenetic Tree Analysis

**Figure 5:**
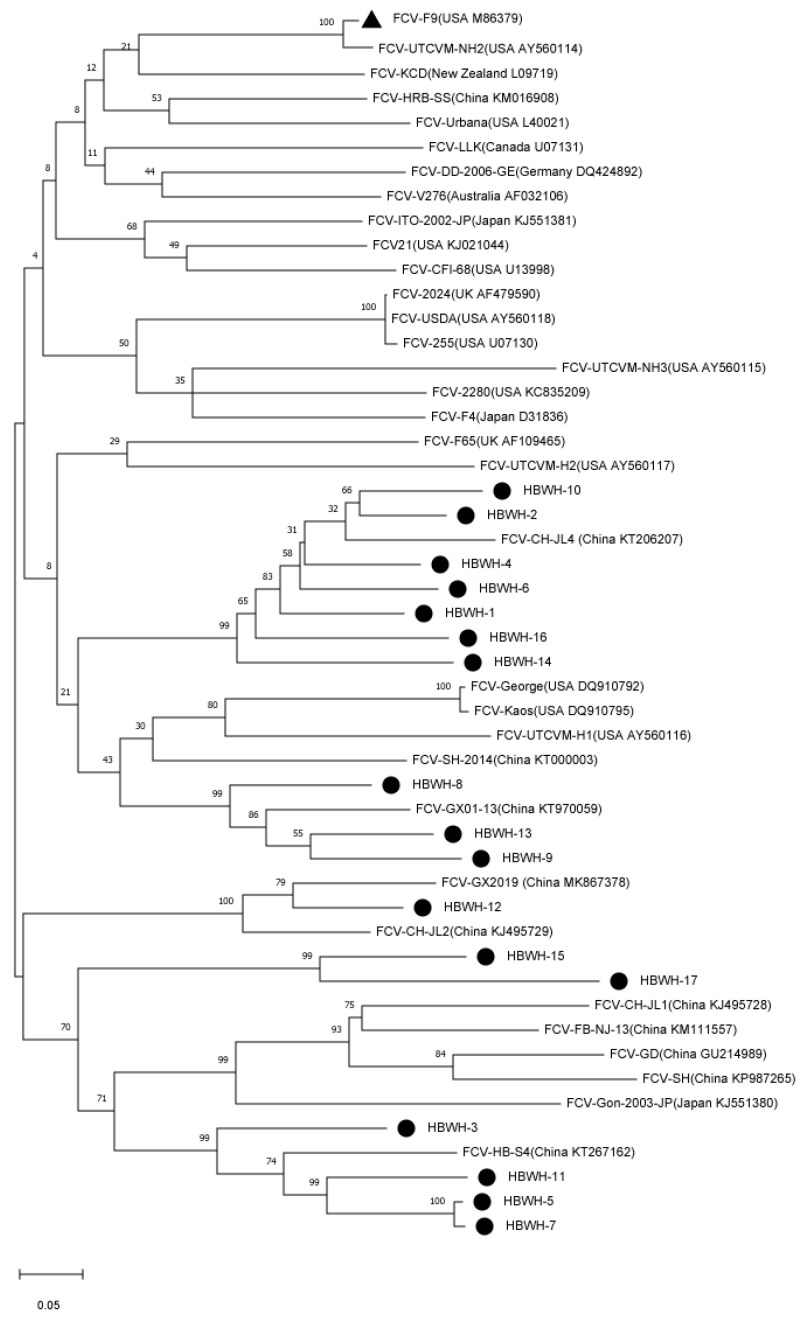
Nucleotide evolutionary tree established by 17 feline calicivirus (FCV) and 34 reference strains. A maximum likelihood (ML) phylogenetic tree was established using the Jones–Taylor–Thornton (JTT) model nucleotide substitution model, 1000 bootstrap replications. Black triangles identify F9-vaccine-associated strains (GenBank No. M86379), and black circles identify 17 isolates in this study.

The phylogenetic tree results showed that the 17 FCV strains in this study were distributed in two clustered topologies. HBWH-1, HBWH-2, HBWH-4, HBWH-6, HBWH-8, HBWH-9, HBWH-10, HBWH-13, HBWH-14, and HBWH-16 are clustered as a clade, and the other 7 strains are clustered in another branch. The two clustered clades have the closest genetic distance to the Chinese strain and were genetically distant from F9-vaccine-associated strains (GenBank No. M86379). However, these FCV-infected cats were immunized with the F9 vaccine (Figure 5).

## 4. Discussion

Epidemiologically FCV, FHV-1, *M. felis*, and *C. felis* are worldwide distributed upper respiratory disease (URTD) infections [22]. FCV and FHV-1 are responsible for 80–90% URTD cases, while the rest are caused by *M. felis*, *C. felis*, and *Bordetella bronchiseptica* (*B. bronchiseptica*) [26]. These infections among the feline population are important recurrent problems for cat owners and veterinarians globally. In the current study, a total of 1158 cat samples with clinical symptoms were collected from Wuhan province, China. A total of 465 (40.2%) FCV positive samples and 180 FHV-1 positive samples (15.5%) were analyzed through PCR/RT-PCR detection. The FCV positive rate was much higher than that of FHV-1 positive samples. The reason may be the application of vaccines. Studies have shown that vaccines are likely to reduce the number of cats shedding FHV-1 but have no significant effect on the number of cats shedding FCV; because FCV is an RNA virus with a wide range of genetics and antigenic diversity, while FHV-1 is a stable DNA virus [27,28,29]. The third factor that might account for such a large range is the testing protocol, which can differ based on the presence or absence of an infection, i.e., the population being tested, the kind of specimen, and the technique of testing [30]. The low prevalence of FHV-1 may also be attributable to the chronicity of the disease in cats [31,32]; it was found to be 15.5% with fewer symptoms than FCV.

The incidence of infection with all of the upper respiratory tract infections listed above was more remarkable in male cats (toms) that had not been neutered or spayed compared to the rate of infection in female cats. This disparity in the incidence of infection might be attributable to the fact that male cats spend more time outside and are thus more likely to come into contact with infectious pathogens [33,34,35]. Another study represents no difference in the rate of URTD based on gender in sheltered cats [16]. Previous work on privately owned cats shows that the rate of FHV-1 in male cats was significantly higher than that of the opposite gender [13]. The incidence of URTD in younger cats was significantly higher than in adults. These findings were in line with a previous study [14]. Moreover, the results were consistent with an earlier study, even in sheltered and stray cats [33]. The most probable reasons would be the lower immune status of kittens and difficulty managing growth, which made them more susceptible to RTD infections [33]. British shorthair cats accounted for the most significant proportion of breeds, with 38% cases, and the most minor ratio was Garfield, with 3% cases. According to Tran et al., purebred cats or purebred crosses are more prone to URTD than mixed breeds [36]. Purebred cats experience more stress levels due to their genetic influence [37], but the exact association still needs further investigation. Moreover, other factors such as sheltered or privately owned, gender, individual causative agent, and nutritional status should also be considered to determine the exact mechanism. 

Our current statistics on the immune status show that the proportion of cats with complete vaccination was more protective (62%) compared to non-vaccinated cats (37%). However, it is also essential to know that the protection does not refer to complete immunization. Hence, the vaccine of FHV-1 and FCV does not provide sterilizing immunity but can reduce the disease’s incidence and severity, as described by [38]. Moreover, the analysis of clinical signs in our study showed that the feline calicivirus infection was associated with oral symptoms, feline herpesvirus, and mycoplasma feline infection cases mainly showed sneezing, while conjunctivitis was the main symptom of feline chlamydial infection. All these clinical signs are similar, as observed in a previous study [9]. Moreover, it has been revealed that the frequent high fever and corneal ulcers in FHV-1, and FCV refer to throat, tongue, and palate ulcers [32]. Clinically, URTD is linked with various symptoms, but none can be singled out as the pathognomic indicator of a specific infection. The incidence of FCV, FHV-1, *M. felis*, and *C. felis* infection in different seasons was analyzed, and the overall positive detection rate of infection was 64.3%. The infection rate of FCV was the highest in autumn (45.5%), while the upper respiratory tract infection in autumn was recorded at 66.9%, especially in the rainy season. However, there was no statistical significance noticed (*p* > 0.05). The same findings were observed in a previous study [39].

FCV has confirmed the cross-reactivity of vaccine strains. Cats completely vaccinated with feline triplets still had a high infection rate, probably due to altered antigenic sites [40,41]. VP1 is the most mutated gene in the FCV virus genome, including six regions A–F. These six regions contain three antigenic epitopes, essential targets for virus-neutralizing antibody binding [42,43]. VP1 is an important target for clinical diagnosis and vaccine development and a gene for phylogenetic tree construction [44,45]. The FCV isolated in this study is relatively close in genetic distance to the previously reported isolates in China and far from the vaccine-related strain F9, which may lead to the inability of the F9 vaccine to provide effective immune cross-reaction. Based on the analysis of VP1 AA, the sequence of the VP1 E region has the effect of binding to cellular receptors and can distinguish the virulence of FCV [46]. The amino acid sequence NGT (Asn-Gly-Thr) at positions 440–443 (439–441 in the F9 strain) in the E-5’HVR has strong antibody reactivity [47]. However, the FCV in this study is not consistent with F9. More interestingly, the HBW10 strain has T442 insertion, and the amino acid sequence at 447–459 is not compatible with F9, which may lead to the occurrence of the antigenic epitope, resulting in immune failure. There are some limitations related to the current study. For example, the collection of samples was limited to a certain location. Such a compilation of data would have little use. However, results are essential for future epidemiological studies because they boost the chances of identifying new targets for region based vaccination.

## 5. Conclusions

Male cats, especially young cats (up to 12 months), are more susceptible to upper respiratory tract infection in Wuhan, China. Cats fully immunized with the feline triple vaccine are still at a high risk of having a condition of FCV with stomatitis at a 40.2% infection rate. Moreover, our current study found FCV was genetically different from the vaccine-associated F9 strain in Wuhan, China, urging the development of a vaccine for the local FCV strain. The findings give a foundation and theoretical reference for creating such strain-specific vaccinations to prevent and cure upper respiratory tract infections, not only in an area but across the whole country.

## Figures and Tables

**Table 1:** The PCR primers involved in this study.

Primer	Sequence (5’ to 3’)	Size	References
FHV-1 TK gene	F:GACGTGGTGAATTATC	288 bp	[19]
	R:CAACTAGATTTCCACCAGGA		
FCV VP2 gene	F:TGGTGATGATGAATGGGCATC	477 bp	This study.
	R:ACACCAGAGCCAGAGATAGA		
M. Felis 28S gene	F:CGCTAATAGGGAATGTGAGCTAGG	121 bp	[20]
	R:TGTCTGAACCTCCAGTTTCTCTGG		
C. felis OMP2 gene	F:ATG TCC AAA CTC ATC AGA CGA G	590 bp	[21]
	R:CCT TCT TTA AGA GGT TTT ACC CA		
FCV	F:TTCGGCCGTTTGTCTTCC	679 bp	[22]
	R:TTGTGAATTAAAGACATCAATAGACCT		

Note: F is forward primer, R is reverse primer.

**Table 2:** Total positive rates of FCV, FHV-1, M. felis and C. felis with upper respiratory tract infection in Wuhan, China.

Seasons *	No. of Positive Cats/Tested Cats%	NO. of Positive Cats/Tested Cats (%)			
		FCV	FHV-1	*M. felis*	*C. felis*
Spring	170/281 (60.5%)	96/281 (34.2%)	39/281 (13.9%)	70/281 (24.9%)	24/281 (8.5%)
Summer	135/219 (61.6%)	83/219 (37.9%)	18/219 (8.2%)	53/219 (24.2%)	21/219 (9.6%)
Autumn	200/299 (66.9%)	136/299 (45.5%)	41/299 (13.7%)	90/299 (30.1%)	23/299 (7.7%)
Winter	239/359 (66.6%)	150/359 (41.8%)	82/359 (22.8%)	98/359 (27.3%)	17/359 (4.7%)
Total	744/1158 (64.3%)	465/1158 (40.2%)	180/1158 (15.5%)	311/1158 (26.9%)	85/1158 (7.3%)

* Spring (March to April), summer (May to August), autumn (September to October), winter (November to March).

**Table 3:** Comparison of Nt and AA homology between FCV strains with reference strains. All data were presented in percentages.

	Reference Strain	F9 Vaccine	China	USA	UK	Japan
Isolates	nt	69.3–73.1	67.3–84.4	68.6–76.6	69.3–74.5	68.7–74.8
	AA	91.8–93.8	90–96.7	90.3–94.8	90.9–93.9	90.5–94.7

## Data Availability

The data that support the findings of this study are available on request from the corresponding author.
